# Overcoming the data limitations in landslide susceptibility modeling

**DOI:** 10.1126/sciadv.adt1541

**Published:** 2025-02-21

**Authors:** Jacob B. Woodard, Benjamin B. Mirus

**Affiliations:** U.S. Geological Survey, Geologic Hazards Science Center, 1711 Illinois Street, Golden, CO 80401, USA.

## Abstract

Data-driven models widely used for assessing landslide susceptibility are severely limited by the landslide and environmental data needed to create them. They rely on inventories of past landslide locations, which are difficult to collect and often nonrepresentative. Furthermore, susceptibility maps are most needed in regions without the means to assemble an inventory. To overcome these challenges, we develop a method for assessing shallow landslide susceptibility based on a probabilistic morphometric analysis of the landscape’s topography, rather than the characteristics of landslides. The model assumes that hillslopes with higher relief and gradient compared to the surrounding landscape are more prone to landslides. We demonstrate the superior performance of this approach over contrasting data-driven models across the northwestern United States. As our morphometric model only requires elevation data, it overcomes the major limitations of data-driven models and facilitates the creation of effective susceptibility models in areas where it was previously unfeasible.

## Teaser

Reliable landslide susceptibility maps can be developed in areas without landslide data.

## INTRODUCTION

Landslides occur around the world (*1*), affecting lives, infrastructure, and property. In the United States alone, landslides are estimated to cause billions of dollars in damages every year (*2*, *3*). With current trends in climate change and urban expansion into less stable terrain, losses associated with landslides are expected to increase (*1*, *4*–*7*). The effects of these losses are greater in more vulnerable populations (*8*–*10*). However, vulnerable nations or states generally have fewer resources to develop tools for mitigating landslide-related losses. This presents a predicament, as the regions that have the most need for landslide mitigation products generally have the fewest resources for creating them.

Regional-scale (>100 km^2^) landslide susceptibility maps show the likelihood of a location experiencing a landslide and are among the most fundamental tools for mitigating the impacts of landslides. Regional-scale products provide consistent assessments across scales important for developing infrastructure plans (*11*), disaster relief planning (*12*, *13*), and informing where more detailed studies should be carried out (*14*). Common approaches for creating regional susceptibility maps include using physically based models (*15*), heuristic approaches (*16*), and data-driven statistical models (*17*). Physically based models aim to characterize the driving and resistive stresses within the hillslope by explicitly accounting for its geotechnical parameters (*18*). As such, this approach requires detailed geotechnical data that are not easily obtained over regional scales (*15*, *19*, *20*). Without these data, assumptions about the spatial distribution of geotechnical properties must be made that can nullify the benefits of a physically based model (*21*, *22*). Consequently, it is generally impractical to use a physically based model over large regions. In contrast, heuristic models assess susceptibility using expert opinion on the most important environmental attributes (e.g., slope, geology, and vegetation) and how these attributes control slope failure. However, the heuristic model’s reliance on the developer’s opinions makes their results inherently biased, inconsistent, and difficult to reproduce (*23*). Last, data-driven statistical models use the environmental attributes of known landslide and nonlandslide locations to train a statistical model to determine the probability of a landslide occurring at a location given its local attributes. These models require an inventory that reliably shows locations with and without evidence of landsliding and representative geospatial databases of the environmental attributes to be meaningful. Because of the proliferation of pertinent geospatial datasets and the relative ease of producing useful results, data-driven models have gained much attention over the last decades (*17*).

Despite extensive progress on developing methods of optimizing data-driven susceptibility models with landslide inventories (*24*–*27*), the inherent biases of the inventories and imperfect environmental data commonly used to develop the models can limit their application to regions where landslide processes are already well characterized. Landslide inventories do not exist in most areas of the world (*28*–*31*), and efforts to extrapolate data-driven models to data-sparse regions generally produce unreliable results (*27*). In areas where landslide inventories do exist, they rarely include a temporal component. Rather, teams will map any area within the modeling domain with evidence of landsliding manifest by arcuate head scarps, hummocky topography, or disturbed vegetation (*32*, *33*). Hence, these inventories are an amalgamation of many landslide events through time and are often referred to as geomorphological inventories (*34*). However, evidence of shallow landsliding generally does not persist on the landscape as long as it does for large deep-seated landslides. As a result, most geomorphological landslide inventories are biased toward including a lower ratio of shallow landslides to deeper-seated landslides than the ratio expected from an event-based inventory (*35*). In contrast, event-based inventories that capture more shallow landslides (*34*, *36*) generally do not show the distribution of landslides over time. This is due to the considerable expense of continually resurveying the study site for new landslides. Datasets that cover multiple landslide events over the same region are exceptionally rare (*37*–*40*). As a result, most inventories and data-driven models are biased toward characterizing large deep-seated landslides that occur much less often than small shallow landslides (*34*, *36*) and are generally less destructive (*10*). Furthermore, the detailed environmental datasets required for building these models are often too coarse and imprecise to effectively differentiate the factors that exert localized control on landsliding for individual hillslopes. This requires modelers to either assess susceptibility using large mapping units that overgeneralize the level of landslide potential or extrapolate their data and risk misrepresenting the true environmental conditions. To date, methods that can accurately assess susceptibility require detailed environmental data and high-quality landslide inventories; in this respect, innovations in susceptibility modeling to overcome these data challenges are needed.

A less used method of assessing susceptibility is through morphometric models that analyze the local terrain to infer the limits of stable topography. Culmann (*41*) laid the foundation of this method by deriving an equation for the maximum stable relief (*H*_c_) an idealized hillslope can obtain given its mean slope (β_1D_) and soil’s unit weight (γ), cohesion (*c*), and angle of internal friction (ϕ) as follows

$$H_{c}=\frac{4c}{\gamma }\frac{\text{sin}\beta_{1D}\text{cosϕ}}{\left[1-\text{cos}\left(\beta_{1D}-\phi \right)\right]}$$ (1)

Substituting the critical hillslope length ($L_{c}=H_{c}/\text{sin}\beta_{1D}$) for *H*_c_, Eq. 1 becomes

$$L_{c}=\frac{4c}{\gamma }\frac{\text{cosϕ}}{\left[1-\text{cos}\left(\beta_{1D}-\phi \right)\right]}$$ (2)

The basis of these equations is that the driving stresses acting on the hillslope can only reach a certain threshold before exceeding the local resistive stresses and initiating a slope failure. It follows that the current hillslope length and slope can provide insights into the effective strength of the underlying earth materials. The method assumes the terrain is strength-limited (i.e., the landscape is prone to landsliding) and that any landslide results in a complete failure of the hillslope. As a result, this method is only appropriate for shallow landslides, which are typically translational failures in the upper few meters of regolith (*42*, *43*). With these reasonable assumptions, Eq. 2 indicates that the local distributions of hillslope length and slope will reach an upper limit that is controlled by its effective strength, which may include both the material strength and apparent influence of other factors such as vegetation. We can use these limits as a metric for shallow landslide susceptibility (*44*). Many studies have used a similar approach of analyzing the landscape topography to estimate the limits of stable terrain (*20*, *44*–*52*). However, no study we are aware of has used this theory as a method of assessing susceptibility independent of landslide inventories.

**Fig. 1.**
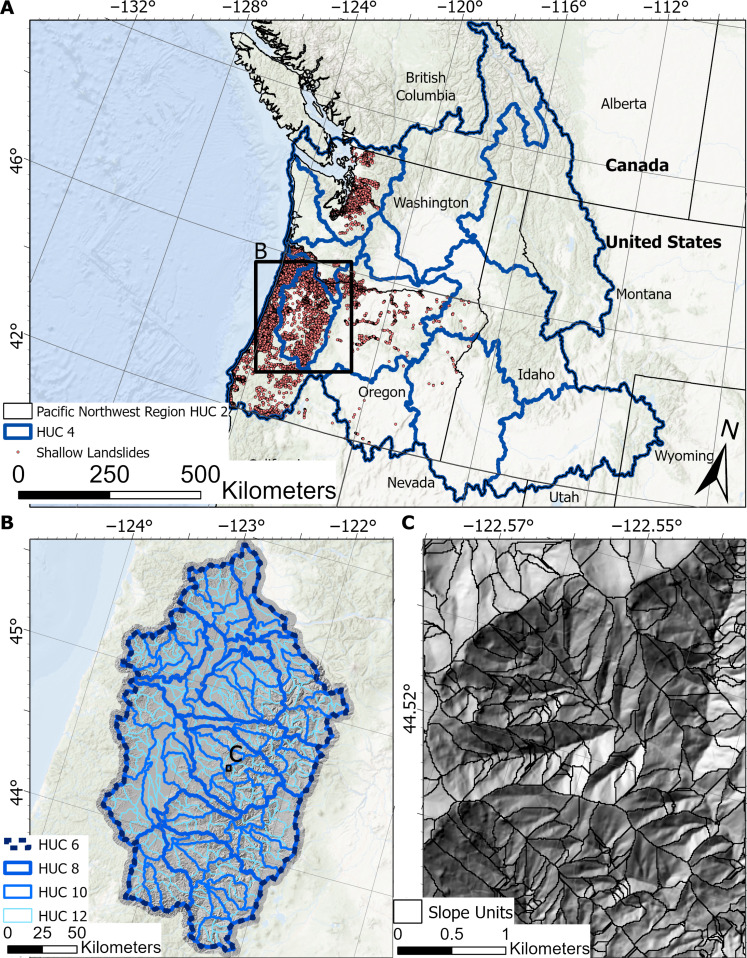
Overview of study area. (**A**) Study area covering the Pacific Northwest regional watershed with the 25,660 mapped shallow landslide points (*76*, *95*, *96*) and the hydrologic unit code (HUC) 4 watersheds. (**B**) The HUC 4 Willamette watershed along with its subwatersheds (HUC 6, 8, 10, and 12). (**C**) The raster we use as our slope units. Basemaps for (A) and (B) are from the ESRI World Hillshade (*105*) and World Oceans (*106*). Shaded relief map in (C) is from the NHDPlus HR dataset. Black boxes in (A) and (B) show the extents of (B) and (C), respectively.

In this study, we develop a statistical morphometric model that can generate estimates of landslide susceptibility for shallow landslides using only elevation data. The model is based on the fundamental principle that as the slope and size of a hillslope increase, the relative probability of failure increases due to the associated increase in driving stress. We contrast the morphometric model to two data-driven susceptibility models over the northwestern United States (Fig. 1): a logistic regression machine learning model used in previous studies (*27*) and a parsimonious national susceptibility model (*53*). We first contrast the morphometric and logistic regression model over the Willamette watershed in western Oregon (Fig. 1B). Next, we contrast the national susceptibility model and the morphometric model over the Pacific Northwest Region (Fig. 1A). Both areas are geologically, ecologically, and topographically diverse and provide ideal locations for testing the effectiveness of the different modeling approaches. We demonstrate that the morphometric model’s performance is generally better than the data-driven models, despite the lack of landslide occurrence data or complex environmental predictors used in model parameterization. Because of the proliferation of remote sensed elevation data (*54*), our morphometric approach can be used to effectively and equitably communicate the landslide potential anywhere in the world.

## RESULTS

### Development of a morphometric susceptibility model

**Fig. 2.**
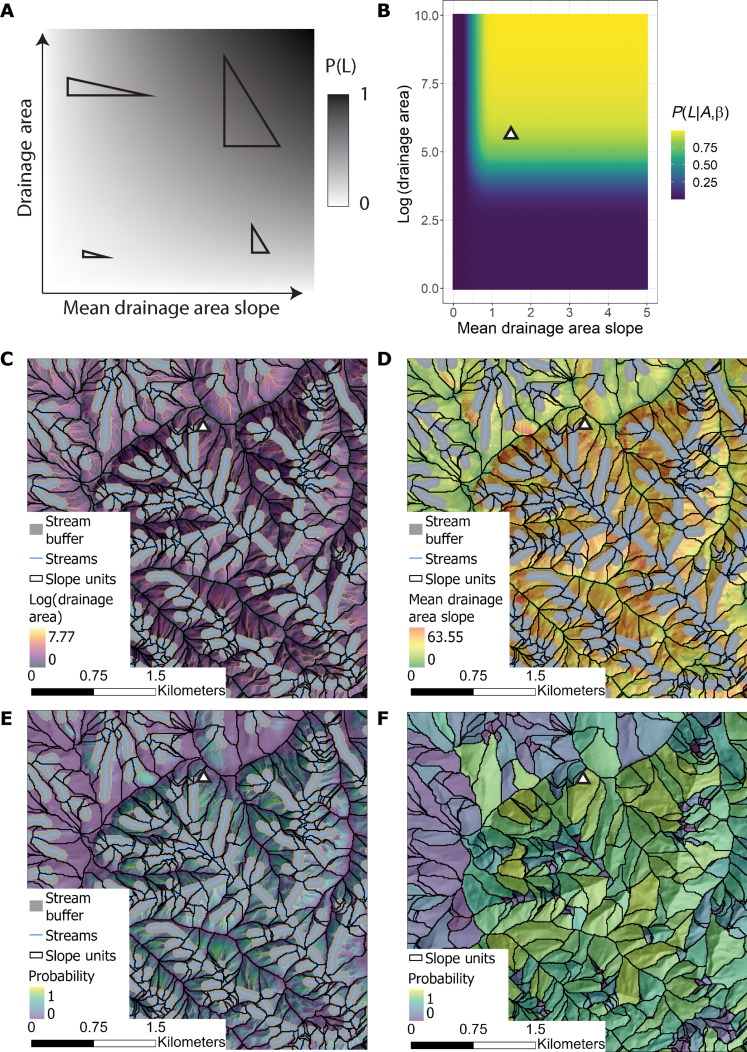
Morphometric susceptibility model workflow. (**A**) Two-dimensional modeling scheme used to assess landslide susceptibility. Increasing the area or slope of the hillslope increases the probability of a failure occurring. Black-right triangles show two-dimensional representations of different hillslope geometries relative to landslide probability. (**B**) A joint cumulative distribution function is calculated using (**C**) the drainage area and (**D**) the mean drainage area slope to estimate (**E**) the probability of shallow landslide occurrence at the raster scale. (**F**) The maximum value of the probability raster in (E) is calculated for each slope unit to produce the final susceptibility map. (C) to (F) have the same extent as Fig. 1C.

We develop the morphometric model that expresses the probability of a landslide (*P*(*L*)) from a statistical analysis of the local distributions of slope and hillslope area (Fig. 2). Fundamentally, this method is based on the theory introduced in Eq. 2 that the driving stress acting on a hillslope increases with the size and slope of the surface. Assuming a uniform resistive stress across the domain of interest, we can formalize this theory in probability space as follows

P(L∣β)∝β (3)

P(L∣A)∝A (4)

where β is the average drainage area slope of the surface and *A* is the drainage area. Slope is generally considered the most important environmental variable contributing to shallow landsliding due to its direct effect on driving stress (*18*, *55*). Hillslope size, which is approximated by *A*, also directly affects driving stress if the failure plane spans the length of the hillslope, as assumed in Eq. 2. Further justification for Eq.4 is found in the statistical principle that as the sample size increases, the likelihood of capturing an event increases. In addition, many studies have indicated that landsliding increases where hillslopes are larger (*56*–*60*) due to increased hydrologic forcing (*61*) and driving stress potential (*62*). Thus, Eqs. 3 and 4 have firm foundations in the principles of soil mechanics and statistics if we assume a constant resistive stress. However, resistive stresses are not uniform over regional scales. Next, we introduce how we can constrain the resistive stresses over a domain by analyzing the local distributions of β and *A*.

As described by Eq. 2, if we assume that the domain is strength limited, then the local distributions of *A* and β can provide insights into the resistive stresses acting on the hillslopes. Furthermore, analyzing the current topography can elucidate the environmental conditions not explicitly accounted for in Eq. 2 that contribute to slope failure such as local climate, vegetation, and seismicity (*63*–*66*). Building on Eqs. 3 and 4, we assume that as the values of *A* and β approach the limits of their respective sample spaces within the domain, the probability of landsliding increases proportionately. We formalize this principle as follows

P(L∣A,β)∝P(A≤a,β≤b) (5)

The left-hand side of Eq. 5 represents the conditional probability of landsliding given values of *A* and β. The right-hand side of Eq. 5 is the joint cumulative distribution function and represents the probability that *A* and β will have a value less than or equal to *a* or *b*, respectively. We limit β to values greater than 15° to account for the relatively low potential for shallow landslides to initiate below this value (*44*, *67*–*71*). Refer to the Materials and Methods for how β and *A* are calculated. Importantly, P(L∣A,β) depends on the sample spaces of *A* and β. That is, the domain where the method is applied. In this way, the model can account for variations in landslide initiation conditions unique to the local environment.

Selecting the appropriate scale of the domain is a compromise between accounting for the unique local conditions that contribute to landsliding and the fact that not all topographies are strength limited. For example, applying Eq. 5 to a relatively flat region that has no evidence of landsliding would output a susceptibility map that indicates some areas as being susceptible that are not. In contrast, applying Eq. 5 to an entire continent would only map the portions of the terrain that have the largest and steepest hillslopes as susceptible, omitting regions that have lower resistive stresses and can experience failures at lower values of β and *A*. Furthermore, topography and geological properties are often described as scale invariant (*72*). Consequently, likely no spatial threshold is ideal for applying Eq. 5. To help overcome this challenge, we develop a stratified model where Eq. 5 is applied at multiple scales and these outputs are combined into one final model as follows

$$P_{s}\left(L\right)=\frac{1}{\sum_{i=1}^{i=n}W_{i}}\left[W_{1}P_{1}\left(L|A,\beta \right)+\cdots +W_{n}P_{n}\left(L|A,\beta \right)\right]$$ (6)

where $W_{i}$ is the weight of the domain used to calculate the final landslide probability [$P_{s}\left(L\right)$]. Any number of domain divisions and weights can be used for calculating $P_{s}\left(L\right)$. Here, we select the largest scale to be the Pacific Northwest Region hydrologic unit code (HUC) 2 watershed (Fig. 1A) and then create a model of $P_{i}\left(L\right)$ for each HUC watershed within the HUC 2 watershed down to the HUC 12 watersheds (Fig. 1). We also evaluate two different weighting schemes. The first uses a unit weight ($W_{i}=1$). The second uses the area of the domain used to calculate $P_{i}\left(L\right)$. The area weight considers the different scales for determining failure, favoring the larger watershed values that guarantee the inclusion of strength-limited topography, while still accounting for local terrain conditions that are more likely to have similar resistive strengths (*73*).

We display our final susceptibility maps using slope units, which allow us to display the susceptibility results over the hillslope scale (Fig. 2F). This is appropriate due to the use of β and *A* in Eq. 5, which are designed to characterize the entire slope rather than a single raster grid cell. Slope units segregate the terrain into individual hillslopes by splitting the domain by drainage and divide lines. We measure the maximum values of $P_{s}\left(L\right)$ within each slope unit so the map displays the probability that a landslide occurs within a given mapping unit, consistent with the most common definition for susceptibility (*74*).

### Comparison between the morphometric and logistic regression model

Logistic regression is the most commonly used machine learning method for landslide susceptibility (*17*) due to its ease of use and its tendency to not overfit the input data (refer to Materials and Methods) (*26*). We limited the terrain attributes used to train the model to some of the most commonly informative attributes for predicting landslide susceptibility (*55*) that can be derived from the U.S. Geological Survey’s three-dimensional elevation program (3DEP) dataset (*75*), namely, elevation, slope, aspect, roughness, and curvature (refer to Materials and Methods). Limiting the terrain attributes to those derived from the 3DEP dataset mitigates potential biases common in other datasets, such as geology or vegetation cover. We develop a susceptibility model over the HUC 4 Willamette watershed in Oregon (Fig. 1) using their landslide inventories from the Oregon Department of Geology and Mineral Industries (*76*) (refer to Materials and Methods). We further processed the inventories to include only shallow landslides using a failure plane depth threshold of 4.6 m. We limit this analysis to the Willamette watershed due to the relative abundance of shallow landslide locations (*n* = 8363, or 33% of the data available over the Pacific Northwest regional watershed), which benefits the data-driven approach by reducing the chances of incorrectly labeling the mapping units as containing or not containing evidence of landsliding. The sampling ratio of event to nonevent data points can have large effects on a model’s performance (*77*, *78*). To account for these effects, we develop two different models: one that uses a 1:1 sampling scheme of landslide to nonlandslide data [LR(1:1)] and another that samples all the data within the training data [LR(all)]. The LR(all) model has a sampling ratio of 1:33. We compare the performance of the logistic regression models to the morphometric model using a spatial clustering cross-validation scheme that evaluates how the model would perform on a new location and comparing the final susceptibility maps (refer to Materials and Methods).

The most common metrics for quantifying the quality of susceptibility models measure how well the model estimates where landslides have and have not occurred as determined by the landslide inventory (e.g., receiver operator characteristics and Brier score) (*17*). However, over large regions where the landslide inventories are known to be incomplete, these metrics may be inappropriate due to the high potential of falsely categorizing a mapping unit as not containing a landslide. Consequently, we introduce a modified earth moving distance (EMD) metric (*79*) that quantifies model performance based on its ability to successfully characterize known landslide areas while minimizing the area of the domain with elevated susceptibility values (refer to Materials and Methods). Similar indicators have been used for evaluating landslide susceptibility models over regions with known data shortages (*53*, *80*). Higher positive EMD values indicate better model performance. We also measure performance using the Brier score, which calculates the mean square error between the observed landslide data (a binary variable of landslide existence) and the model predictions (refer to Materials and Methods). Lower Brier scores indicate better model performance.

**Fig. 3.**
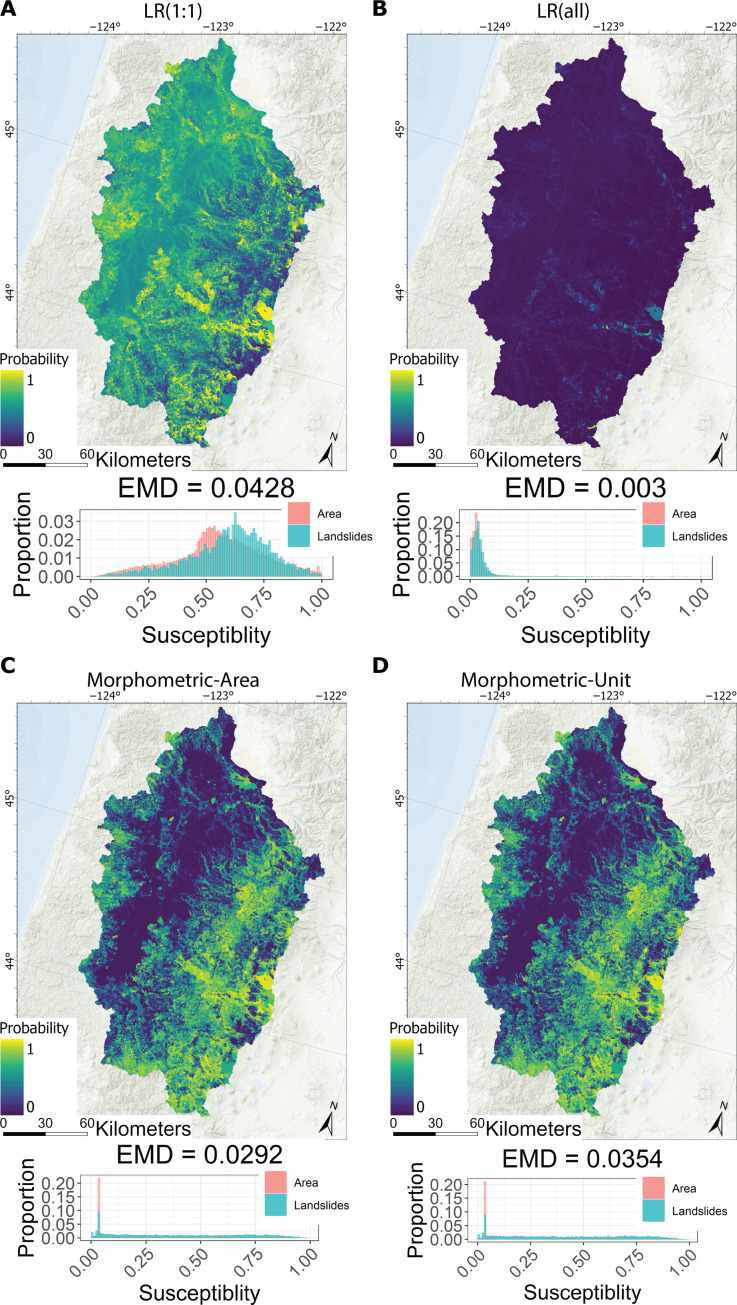
Comparing results of logistic regression and morphometric models. Susceptibility results over the HUC 4 Willamette watershed from (**A**) the logistic regression model using a 1:1 sampling ratio, (**B**) the logistic regression model using all the available data, (**C**) the morphometric model using area as the weights in Eq. 6, and (**D**) the morphometric model using unit weights. Lower histograms show the results of the EMD metric for all the data within the domain. Note the different scales of the *y* axes between the histograms. Basemaps are from the ESRI World Hillshade (*105*) and World Oceans (*106*).

**Fig. 4.**
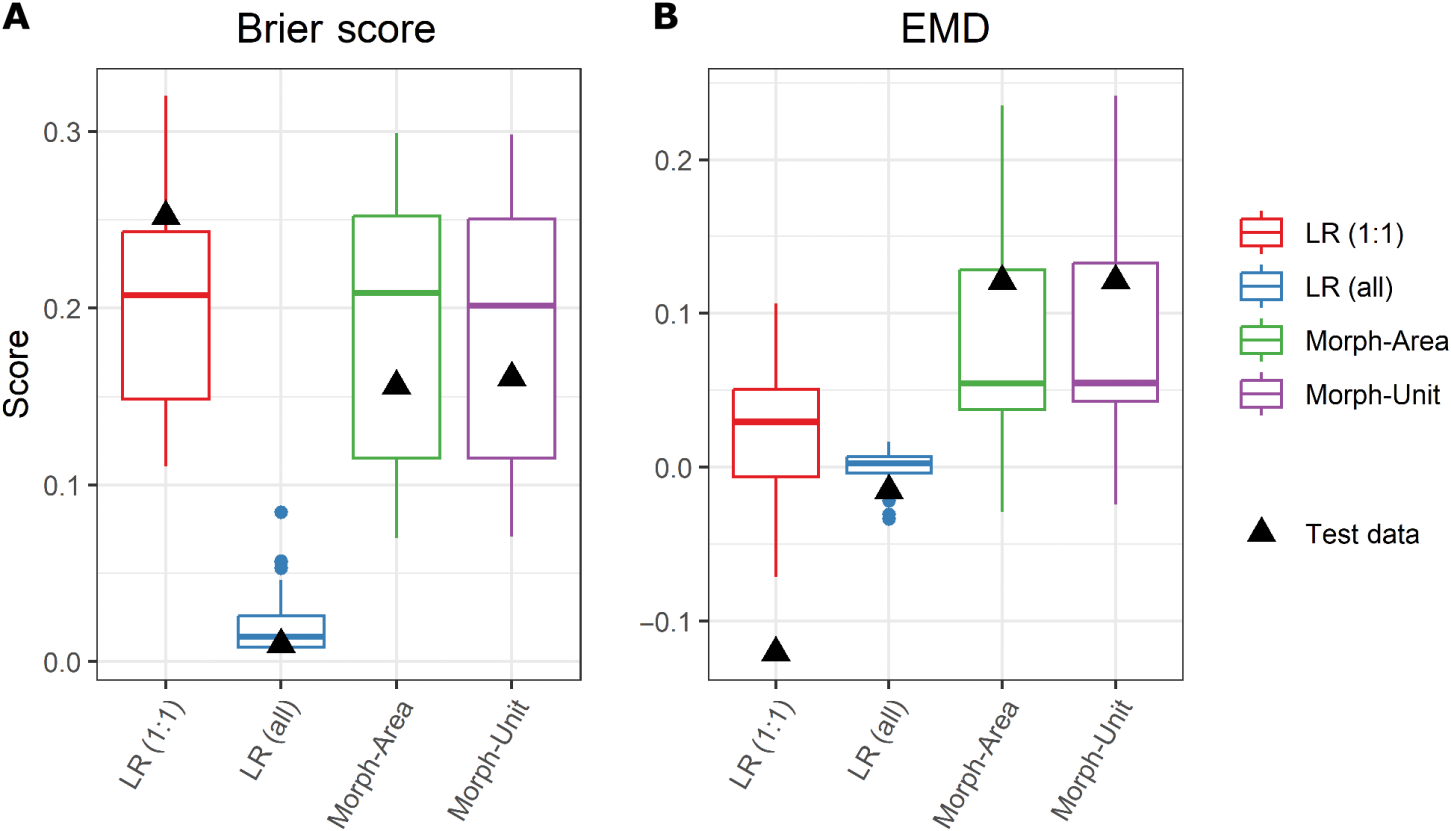
Logistic regression and morphometric cross-validation over the Willamette watershed. Box plots of the spatial cross-validation results over the HUC 4 Willamette watershed using (**A**) the Brier score and (**B**) the EMD. Different colors correspond with the model being evaluated: logistic regression using a 1:1 sampling ratio [LR(1:1)], logistic regression using all the data [LR(all)], the morphometric model using area weights (Morph-Area), and the morphometric using unit weight (Morph-Unit). Boxes show the interquartile range, the horizontal line is the sample median, the bars extend to 1.5 times the interquartile range above the box plot, and the dots represent data beyond the bars. Black triangles show the metrics for the test datasets.

The morphometric model generally better characterizes the level of susceptibility compared to the logistic regression models, even in a region with relatively abundant data. Figure 3 shows the logistic regression and morphometric model results over the Willamette watershed. Most of the areas in the LR(1:1) model have probabilities between 0.5 and 0.75. In contrast, most of the area in the LR(all) has values near zero due to the increase in the nonlandslide data used to train the model. The two morphometric models are very similar in their final model outputs and model metrics, indicating that the weighting scheme has little effect on model behavior. Compared to the logistic regression models, the morphometric susceptibility values are more spread out with a slight clustering near zero. The EMD values of the final maps over the Willamette watershed evaluated using all the available landslide data within this domain indicate that the LR(1:1) model performs the best with a value of 0.043, followed by the two morphometric models with values of 0.029 and 0.035, and the LR(all) model performs the worst with a value of 0.003 (Fig. 3). Because these EMD values are calculated using all the data within the Willamette watershed, the logistic regression results are inflated compared to the cross-validation results (Fig. 4).

The cross-validation procedure indicates that the morphometric models are the most transferrable. Figure 4 shows the Brier and EMD scores from the cross-validation procedure. The LR(all) model has the best Brier scores but the worst EMD values. The good Brier score is due to most of the slope units not containing mapped landslides and the LR(all) model mapping most of the slope units with low probabilities. However, the inability of the LR(all) model to map areas with existing landslides as susceptibility results in the worst EMD value. The LR(1:1) has a Brier score comparable to the two morphometric models but a worse EMD value. The two morphometric models generally show the best EMD values. The test datasets (black triangles) generally show a poorer model score compared to the average cross-validation results for the logistic regression models whereas the morphometric models generally show an above-average performance.

### Comparison between the morphometric and the parsimonious national susceptibility model

The national landslide susceptibility model by Mirus *et al.* (*53*) builds on the theoretical advances by Schmidt and Montgomery (*44*) and a prototype application developed by Godt *et al.* (*81*) for determining areas with some or no potential for landsliding. The national model fits a nonlinear quantile regression curve (*80*, *82*) to the measured slope and relief (using a 100-m moving window) of landslides within the U.S. Geological Survey landslide compilation dataset (*83*) such that 10% of the landslide observations fall below the curve. Nonsusceptible pixels are those whose measured slope and relief fall below the calculated threshold curve, whereas susceptible pixels are those with values above the curve. Mirus *et al.* (*53*) conducted the analysis using 613,724 landslide locations across the United States and the 3DEP elevation dataset. This resulted in a model that categorized more than 99% of observed landslides as having some susceptibility. They did not attempt to discriminate landslides by depth or type. They also converted the binary model into a susceptibility model with continuous integer values ranging between 0 and 81 by downsampling the 10-m model output to 90 m and counting the number of 10-m cells categorized as having some susceptibility within each 90-m cell. To compare the morphometric model to the national model, we convert the original grid-based national model to a slope unit model by taking the maximum raster value present within each slope unit, following the procedure used for the morphometric model. We then rescale the values to a range of zero to one. We note that these manipulations will increase the area of highly susceptible zones compared to the original grid-based maps. In addition, the morphometric map was clipped to match the same extent as the national model before measuring the EMD metric.

**Fig. 5.**
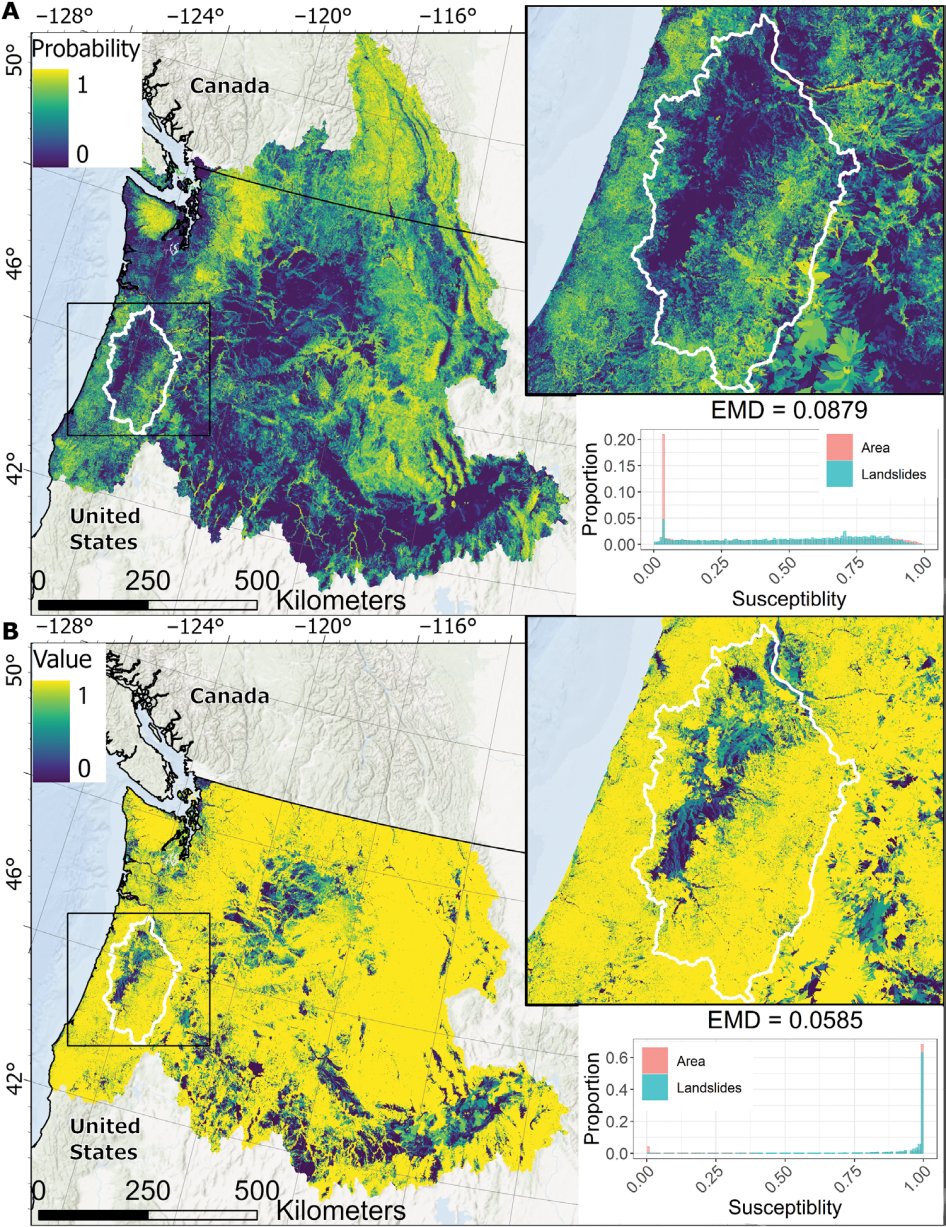
Comparing results of the national and morphometric models. Susceptibility results over the HUC 2 Pacific Northwest Region watershed from (**A**) the morphometric susceptibility model using area weight and (**B**) the national susceptibility model. Inset maps show the Willamette watershed susceptibility models covering the same extent as Fig. 3. Histograms show the results of the EMD metric for the areas within the United States. Note that the national susceptibility model is only available over the United States, not Canada, and that the scale of the *y* axes of the histograms is different. Basemaps are from the ESRI World Hillshade (*105*) and World Oceans (*106*).

The national and morphometric models show notable differences over the Pacific Northwest watersheds (Fig. 5), with the latter having better EMD metrics. The national model has a much larger area with high susceptibility values compared to the morphometric model. In contrast, the morphometric model shows more variability in the susceptibility values. The morphometric model results in a higher EMD value (0.0879) compared to the national model (0.0585), despite the latter being trained on numerous landslides within this region. We do not measure the Brier score over these regions because most of the region has no landslide inventory (Fig. 1), and the national model was developed using the available landslide data. Consequently, the Brier scores would be difficult to interpret. Nor do we conduct a cross-validation comparison as the national model was not developed in this study.

## DISCUSSION

Overall, our morphometric approach is potentially transformative in that it eliminates the need for inventory and environmental data, which are often costly to collect, inconsistent, and lead to model biases. This allows the application of susceptibility modeling in areas that need it the most and yet have the fewest resources for collecting the necessary datasets required by traditional data-driven approaches. Our model metrics indicate that the logistic regression models can have similar model performance over data-rich regions, but they are less transferable compared to the morphometric model. The spatial cross-validation procedure shows similar results between the LR(1:1) and morphometric models (Fig. 4). Also, the LR(1:1) model has the best EMD value when measured using all the data (including data used to train the model) over the Willamette watershed (Fig. 3). However, there is a notable drop in performance of the logistic regression model when evaluated on the test dataset (black triangles in Fig. 4). In contrast, the morphometric models show no decrease in performance between the test and validation dataset evaluations, highlighting their utility in data sparse regions. This is expected because the morphometric model was not trained on any of the landslide data and highlights the high transferability of the morphometric model compared to data-driven techniques. The problem of transferability is a known limitation of data-driven and physically based models (*26*, *27*, *84*). This makes their application questionable anywhere there is not a representative in situ dataset (i.e., landslide inventory and environmental data). The morphometric model overcomes this problem by developing a measure of susceptibility that is independent of these in situ data.

Each model type we evaluated has a different approach for characterizing susceptibility, which is reflected in differences between the final susceptibility maps (Figs. 3 and 5). The primary purpose of the national model is to conservatively identify all areas with any landslide potential by any failure mechanism. The EMD histogram in Fig. 5 indicates that the model is effective at carrying out its designed purpose, as 99.7% of the landslides over the Pacific Northwest have a value greater than zero (i.e., some landslide potential) and most of the landslides have high (>0.5) susceptibility values. However, this good categorization of known landslide locations comes at the expense of an increased proportion of the total area having high susceptibility values. Part of this overprediction may be attributed to the model not discriminating between landslide types like the logistic regression and morphometric models. The logistic regression model tries to identify areas with the same attributes as locations that have already experienced a landslide. Although logistic regression performs well over regions with abundant data, the model’s design to find locations with similar attributes to its training data causes it to perform poorly over regions without a representative landslide inventory (Fig. 4), including most of the northwestern United States. Furthermore, data-driven approaches can only represent the types of hillslopes that have already experienced a failure and is represented in the inventory. This may be neglecting hillslopes that may fail by triggers different from those that caused the landslides in the inventories (e.g., earthquake-triggered landslides) (*85*). In contrast to the data-driven models, the morphometric model attempts to characterize where landslides are likely to occur irrespective of where documented landslides have occurred. By characterizing the morphology of the terrain, the model assigns the highest probabilities to those with the most severe slopes and largest areas. This method of determining susceptibility better characterizes the potential for shallow landslides, even in data-rich regions, than the other two models. In addition, it can capture landslide potential from a wider range of landslide-triggering scenarios compared to data-driven methods.

The two weighting schemes we test for the morphometric model produce similar final outputs. This similarity is likely due to limiting the model to areas with slopes greater than 15°, which acts to remove the influence of relatively flat terrain from the models. Without the influence of the flat terrain, the variance of the slope and drainage area parameters in the different subdomains is reduced. This causes the joint cumulative density functions (Fig. 2B) of the different subdomains to become more uniform.

We compare the morphometric model to data-driven susceptibility models because they are the most used model type for studies covering regional or greater scales (*17*, *27*). There are many approaches for developing data-driven susceptibility models. Exploration of these approaches has been the focus of hundreds of studies over the decades (*17*). We limit our comparison to two approaches that have been used with success at regional or greater scales. Alterations of the modeling algorithm or the data preprocessing could yield different results. However, because of the modest performance benefits these alterations generally produce (*17*, *86*), our analysis provides a reasonable comparison between the data-driven and morphometric models.

Although the morphometric model performs well in our study area, it has some notable drawbacks. The morphometric model is designed to characterize susceptibility at large scales, only showing the general locations where landslides are likely to occur. Hence, if a more localized characterization of landslide potential is desired, alternative methods may be more effective. In addition, the morphometric approach assumes that the whole domain is strength limited. This assumption is valid in regions with relatively high uplift with commensurate erosion rates like portions the Pacific Coast Range (*46*, *87*). However, many regions of the Pacific Northwest may not be prone to landsliding. Restricting the model to slopes greater than 15° and using a stratified model (Eq. 6) helps prevent the model from overpredicting in areas with no potential for landsliding, but it does this at the expense of correctly characterizing the few slope failures that occur on shallower slopes (*53*, *67*–*71*). Application of the model to a strictly non–strength-limited terrain that contains slopes greater than 15° would overpredict landslide potential. In addition, many terrains have heterogenous resistive strengths that may cause steeper terrains to be less prone to landslides compared to adjacent hillslopes covered by weaker material. For example, the Eel River catchment in Northern California is largely composed of mudstone and mélange, which are prone to failure at shallow slopes (mean of 19°), with resistive bedrock blocks that manifest as steep and high relief slopes scattered across the terrain (*87*). In this catchment, the morphometric model may incorrectly label the steep terrain as more susceptible compared to the shallower terrain.

The morphometric model offers a means of creating more objective landslide susceptibility products, which results in a more equitable product for regional landslide loss reduction. An equitable product provides “consistent and systematic fair, just, and impartial treatment of all individuals” (*88*). The morphometric method’s only required input is a digital elevation model (DEM), which is widely available with consistent and unbiased coverages over most of the world. For example, the 3DEP dataset (*75*) provides 10-m elevation data over the entire United States. The Shuttle Radar Topography Mission dataset (*54*) provides 1–arc sec (~30 m) DEMs over most of the world. Limiting our input data for the morphometric model to elevation data overcomes some of the major drawbacks of other susceptibility products caused by inconsistent input data and enables the creation of more equitable susceptibility maps over most of the world.

## MATERIALS AND METHODS

### Slope and drainage area calculation

We use the National Hydrography Dataset Plus High Resolution (NHDPlus HR) (*89*) for measuring the topographic features of interest. The NHDPlus HR dataset aggregates and processes the 3DEP (*75*) elevation data using 22 HUC 4 watersheds that cover the United States. The 3DEP elevation data are 1/3 arc sec (~10 m) resolution elevation rasters with a vertical mean square error of 0.82 m (*90*). We use four rasters within the NHDPlus HR dataset. First, we use the raw elevation raster (elev_cm). Second, we use the HydroDEM raster, which is an altered version of the elev_cm raster that ensures alignment with the HUC watershed boundaries and stream network. It does this by reducing the elevation around the mapped stream network and raising the elevation of the HUC watershed boundaries. Refer to Moore *et al.* (*91*) for details on these alterations. Third, the stream network raster (swnet) shows the locations of all streams evident in the 10 m 3DEP DEMs. Fourth, we use a catchment raster (cat), which is delineated using the uppermost extent of the swnet raster. We use the cat raster as our slope units (Fig. 1). Because we are using slope units to characterize susceptibility, we measure the mean drainage area slope for β, which better characterizes the driving forces over the entire hillslope compared to the local mean slope (Eq. 1). We calculate the mean uphill slope by taking the quotient of the drainage area raster using slope as a weighting factor for each cell and the drainage area raster using a unit weight. We measure the slope using the elev_cm raster and the drainage area using the HydroDEM raster. We use the HydroDEM for the drainage area to facilitate the removal of the observed streams from the raster. This is necessary to avoid inflated drainage area values not representative of hillslopes. The area used in Eq. 5 is the drainage area raster with unit weights. We remove the stream network from all the rasters using the stream network in the swnet raster buffered by 60 m on each side (Fig. 2). This distance is chosen due to how the HydroDEM raster is manipulated to align streams with the DEM (*91*). All processing is carried out in GRASS (*92*) and R (*93*) on the U.S. Geological Survey’s supercomputer Hovenweep (*94*).

### Landslide inventories

We use landslide inventories from the Washington Geological Survey (*95*, *96*) and Oregon Department of Geology and Mineral Industries (DOGAMI) (*76*) to create the logistic regression models and validate all the susceptibility models. The WGS inventory consists of a landslide_deposit and a recent_landslide layer. The landslide_deposit layer includes polygons of landslide features visible in high-resolution (0.9-m) DEMs or their derivatives. The recent_landslide_point layer consists of small or shallow landslides not visible in the DEM but were observed in orthophotos or the field. The landslide point is located at the center of the landslide initiation area. We use the Deposits and Historical_Landslide_Points layers from the DOGAMI inventory. The Deposits layer contains polygons of landslide deposits compiled using a variety of methods and tools for delineating the landslides, including high-resolution (0.9-m) DEMs and their derivatives, orthophotos, and field investigations (*97*). The Historical_Landslide_Points layer consists of landslides from roughly 1923–2023 with the point located near the center of the landslide deposit (*98*, *99*). For both inventories, we limit the polygon datasets to landslides with a known failure depth less than or equal to 4.6 m to omit the large deep-seated landslides abundant in the two inventories. We also assume that most of the historical landslide points are small shallow landslides because these landslide types are the most common in event-based inventories (*34*, *36*). Thus, the logistic regression model will be trained to locate shallow landslides, making it comparable to the morphometric model (*100*). To make the landslide data formats consistent, we convert the landslide polygons to points located at the highest elevation cell within the polygon to approximate the location of the initiation point. In cases of multiple points per polygon, a single point with the highest slope among the highest elevation points is selected as representative. The effects of the different point locations of the DOGOMI Historical_Landslide_Points layer are expect to be largely mitigated due to the use of slope units that are resilient to the effects of minor variations in point locations (*101*). Neither inventory has complete spatial coverage over their respective municipal boundaries. The final combined inventory has 25,660 landslide points (Fig. 1).

### Logistic regression

Logistic regression calculates the log odds of a binary outcome, given some terrain data X as described in the following expression

$$\text{log}\left(\frac{p}{1-p}\right)=\gamma_{o}+\gamma X$$ (7)

where **p** is probability, γ_o_ is an intercept, and γ is a vector of coefficients. The data ***X*** is a matrix containing terrain attributes (predictors) of both landslide and nonlandslide locations. The data’s coefficients are fit to the data using a maximum likelihood criterion. The elevation, slope, aspect (ϕ), roughness, and curvature attributes are all calculated using the elev_cm raster. Roughness is measured as the standard deviation of the elevation using a 100-m square window. Aspect is transformed using cos(ϕ − 45°) to make it periodic and to account for changes in solar heat flux (*102*).

We develop a susceptibility model over the HUC 4 Willamette watershed using the curated inventory and validate the model using a spatial clustering cross-validation scheme. While reserving 30% of the landslide locations as the final test dataset, we use a 10-fold with 10 repeats spatial clustering cross-validation routine on the remaining 70% of the data. The spatial clusters are determined using the K-means algorithm (*103*). This validation procedure helps determine the spatial transferability better than a randomized split (*104*). For the 1:1 sampling ratio [LR(1:1)], nonlandslide locations are randomly selected within the same spatial cluster as the landslide data. For each cross-validation iteration and sampling ratio, the validation dataset consists of all the data in the validation cluster to better capture the models’ ability to identify rare events without inflating the susceptibility values of the whole domain.

### Model metrics

**Fig. 6.**
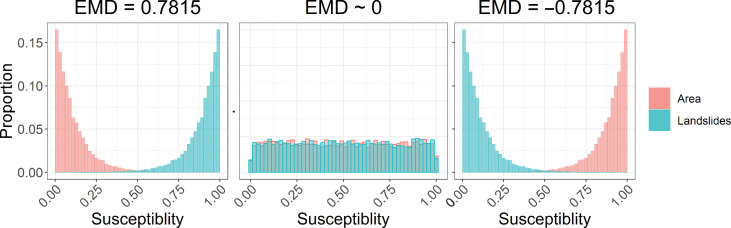
Earth-moving-distance (EMD) metric examples. In cases where the model assigns a high susceptibility value to known landslide locations while the remainder of the domain retains low susceptibility values, a high EMD value is expected. When landslide locations and the rest of the domain have mixed susceptibility values, an EMD value near zero is expected. If the model maps landslides as having low susceptibility values but the rest of the domain has high values, a low EMD is expected.

We use a modified EMD metric (*79*) to quantify model performance. The EMD measures the level of dissimilarity between two distributions by calculating the work (product of height and distance) it takes to move one distribution to overlap with the other. We use the EMD to measure the dissimilarity between a histogram of susceptibility values of known landslide locations and a histogram of the areal extent of susceptibility values (Fig. 6). If we let $Q_{i}$ and $A_{i}$ be the proportion of landslide and area in histogram bin *i*, respectively, then the EMD can be calculated across all *n* bins of width *w* as follows

$$D_{o}=0$$ (8)

$$D_{i+1}=A_{i}+D_{i}-Q_{i}$$ (9)

$$\text{EMD}=w\sum_{i=0}^{n}D_{i}$$ (10)

In contrast to the EMD metric described in Rubner *et al.* (*79*), this method takes into account the direction of the mass movement (Fig. 6). Thus, if the area and landslide probability histograms are perfectly separated with the landslide probabilities above the area probabilities, the model would have an EMD score near one. If the two histograms are perfectly separated but the area proportions are above the landslide probabilities, the model would receive a score near negative one. If the two histograms perfectly overlap, the EMD would be zero. Thus, a well-performing model would produce EMD values closer to one. However, in cases where the inventory is incomplete, a value near one would not be expected because some areas without mapped pixels should have a high susceptibility value. Thus, the EMD metric is best for comparing between models over the same domain. In this analysis, we set the bin width, *w*, to be 0.01. We contrast the EMD to the Brier score, which is a more traditional susceptibility metric that measures the mean-square error between the observed landslide data (*O*; a binary variable of landslide existence) and the model predictions (*P*). The Brier score (*B*) is calculated as follows

$$B=\frac{1}{N}\sum_{i=1}^{N}{\left(P_{i}-O_{i}\right)}^{2}$$ (11)

where *N* is the number of observations. Brier scores range from zero to one with scores closer to zero indicating a better model fit to the observed landslide data.

## References

[1] M. J. Froude, D. N. Petley, Global fatal landslide occurrence from 2004 to 2016. Nat. Hazards Earth Syst. Sci. 18, 2161–2181 (2018).

[2] National Research Council, *Reducing Losses from Landsliding in the United States* (National Academies Press, Washington, 1985).

[3] R. L. Schuster, Socioeconomic significance of landslides, in *Landslides—Investigation and Mitigation. Transportation Research Board Special Report.*, A. K. Turner, R. L. Schuster, Eds. (National Academy Press, Washington, 1996), pp. 12–35.

[4] U. Haque, P. F. da Silva, G. Devoli, J. Pilz, B. Zhao, A. Khaloua, W. Wilopo, P. Andersen, P. Lu, J. Lee, T. Yamamoto, D. Keellings, J.-H. Wu, G. E. Glass, The human cost of global warming: Deadly landslides and their triggers (1995–2014). Sci. Total Environ. 682, 673–684 (2019). 31129549 10.1016/j.scitotenv.2019.03.415

[5] P.R. Shukla, J. Skea, E. Calvo Buendia, V. Masson-Delmot, “Climate change and land: An IPCC special report on climate change, desertification, land degradation, sustainable land management, food security, and greenhouse gas fluxes in terrestrial ecosystems” (IPCC, 2019).

[6] B. Leshchinsky, M. J. Olsen, C. Mohney, K. Glover-Cutter, G. Crook, J. Allan, M. Bunn, M. O’Banion, N. Mathews, Mitigating coastal landslide damage. Science 357, 981–982 (2017). 10.1126/science.aao1722 28883065

[7] A. G. Pendergrass, R. Knutti, The uneven nature of daily precipitation and its change. Geophys. Res. Lett. 45, 11,980–11,988 (2018).

[8] C. A. Dowling, P. M. Santi, Debris flows and their toll on human life: A global analysis of debris-flow fatalities from 1950 to 2011. Nat. Hazards 71, 203–227 (2014).

[9] W. Pollock, J. Wartman, Human vulnerability to landslides. Geohealth 4, e2020GH000287 (2020). 10.1029/2020GH000287 PMC7567151 33094206

[10] P. M. Santi, K. Hewitt, D. F. VanDine, E. Barillas Cruz, Debris-flow impact, vulnerability, and response. Nat. Hazards 56, 371–402 (2011).

[11] B. A. Leshchinsky, A. Booth, M. J. Olsen, N. Matthews, K. Kingen, “Enhanced assessment of projected landslide activity under precipitation and seismicity” (Oregon. Dept. of Transportation. R. Section, 2021); 10.21949/1503647.

[12] Federal Emergency Management Agency, *Local Mitigation Planning Handbook* (FEMA, 2013).

[13] C. Zuzak, M. Mowrer, E. Goodenough, J. Burns, N. Ranalli, J. Rozelle, The National Risk Index: Establishing a nationwide baseline for natural hazard risk in the US. Nat. Hazards 114, 2331–2355 (2022).

[14] J. W. Godt, N. J. Wood, A. B. Pennaz, B. B. Mirus, L. N. Schaefer, S. L. Slaughter, “National strategy for landslide loss reduction” (U.S. Geological Survey Open-File Report 2022-1075, 2022); 10.3133/ofr20221075.

[15] R. L. Baum, D. L. Brien, M. E. Reid, W. H. Schulz, M. J. Tello, Assessing locations susceptible to shallow landslide initiation during prolonged intense rainfall in the Lares, Utuado, and Naranjito municipalities of Puerto Rico. Nat. Hazards Earth Syst. Sci. 24, 1579–1605 (2024).

[16] J.-P. Malet, Y. Thiery, J. Hervás, A. Günther, A. Puissant, G. Grandjean, Landslide susceptibility mapping at 1:1M scale over France: Exploratory results with heuristic model. in *Proceedings of the International Conference on Landslide Processes: From Geomorphologic Mapping to Dynamic Modelling. A tribute to Dr. Theo van Asch*, 315–320 (Strasbourg, France, CERG Editions, 2009), pp. 75–80.

[17] P. Reichenbach, M. Rossi, B. D. Malamud, M. Mihir, F. Guzzetti, A review of statistically-based landslide susceptibility models. Earth Sci. Rev. 180, 60–91 (2018).

[18] T. Lambe, R. Whiteman, *Soil Mechanics* (John Wiley & Sons, New York, 1979).

[19] V. Medina, M. Hürlimann, Z. Guo, A. Lloret, J. Vaunat, Catena Fast physically-based model for rainfall-induced landslide susceptibility assessment at regional scale. Catena 201, 105213 (2021).

[20] M. Bunn, B. Leshchinsky, M. J. Olsen, Geologic trends in shear strength properties inferred through three-dimensional back analysis of landslide inventories. J. Geophys. Res. Earth 125, e2019JF005461 (2020).

[21] A. Carrara, G. Crosta, P. Frattini, Comparing models of debris-flow susceptibility in the alpine environment. Geomorphology 94, 353–378 (2008).

[22] V. Tofani, G. Bicocchi, G. Rossi, S. Segoni, M. D’Ambrosio, N. Casagli, F. Catani, Soil characterization for shallow landslides modeling: A case study in the Northern Apennines (Central Italy). Landslides 14, 755–770 (2017).

[23] F. Fusco, B. B. Mirus, R. L. Baum, D. Calcaterra, P. De Vita, Incorporating the effects of complex soil layering and thickness local variability into distributed landslide susceptibility assessments. Water 13, 713 (2021).

[24] F. Huang, H. Xiong, S.-H. Jiang, C. Yao, X. Fan, F. Catani, Z. Chang, X. Zhou, J. Huang, K. Liu, Modelling landslide susceptibility prediction: A review and construction of semi-supervised imbalanced theory. Earth Sci. Rev. 250, 104700 (2024).

[25] Q. Lin, P. Lima, S. Steger, T. Glade, T. Jiang, J. Zhang, T. Liu, Y. Wang, National-scale data-driven rainfall induced landslide susceptibility mapping for China by accounting for incomplete landslide data. Geosci. Front. 12, 101248 (2021).

[26] S. Steger, A. Brenning, R. Bell, T. Glade, The influence of systematically incomplete shallow landslide inventories on statistical susceptibility models and suggestions for improvements. Landslides 14, 1767–1781 (2017).

[27] J. B. Woodard, B. B. Mirus, M. M. Crawford, D. Or, B. A. Leshchinsky, K. E. Allstadt, N. J. Wood, Mapping landslide susceptibility over large regions with limited data. J. Geophys. Res. Earth 128, e2022JF006810 (2023).

[28] J. Broeckx, M. Vanmaercke, R. Duchateau, J. Poesen, A data-based landslide susceptibility map of Africa. Earth Sci. Rev. 185, 102–121 (2018).

[29] D. B. Kirschbaum, R. Adler, Y. Hong, S. Hill, A. Lerner-Lam, A global landslide catalog for hazard applications: Method, results, and limitations. Nat. Hazards 52, 561–575 (2010).

[30] D. Kirschbaum, T. Stanley, Y. Zhou, Spatial and temporal analysis of a global landslide catalog. Geomorphology 249, 4–15 (2015).

[31] B. B. Mirus, E. S. Jones, R. L. Baum, J. W. Godt, S. Slaughter, M. M. Crawford, J. Lancaster, T. Stanley, D. B. Kirschbaum, W. J. Burns, R. G. Schmitt, K. O. Lindsey, K. M. McCoy, Landslides across the USA: Occurrence, susceptibility, and data limitations. Landslides 17, 2271–2285 (2020).

[32] W. J. Burns, Statewide Landslide Information Database for Oregon, release 3.4 (2017). https://www.oregongeology.org/slido/index.htm.

[33] M. M. Crawford, “Kentucky Geological Survey Landslide Inventory [2023-03]: Kentucky Geological Survey Research Data” (2023); 10.13023/kgs.data.2022.01.

[34] B. D. Malamud, D. L. Turcotte, F. Guzzetti, P. Reichenbach, Landslide inventories and their statistical properties. Earth Surf. Process. Landf. 29, 687–711 (2004).

[35] J. B. Woodard, S. R. LaHusen, B. B. Mirus, K. R. Barnhart, Constraining mean landslide occurrence rates for non-temporal landslide inventories using high-resolution elevation data. J. Geophys. Res. Earth 129, e2024JF007700 (2024).

[36] H. Tanyaş, C. J. van Westen, K. E. Allstadt, R. W. Jibson, Factors controlling landslide frequency–area distributions. Earth Surf. Process. Landf. 44, 900–917 (2019).

[37] K. Bhuyan, H. Tanyaş, L. Nava, S. Puliero, S. R. Meena, M. Floris, C. van Westen, F. Catani, Generating multi-temporal landslide inventories through a general deep transfer learning strategy using HR EO data. Sci. Rep. 13, 162 (2023). 36599911 10.1038/s41598-022-27352-y PMC9813262

[38] J. Samia, A. Temme, A. Bregt, J. Wallinga, F. Guzzetti, F. Ardizzone, M. Rossi, Do landslides follow landslides? Insights in path dependency from a multi-temporal landslide inventory. Landslides 14, 547–558 (2017).

[39] C. Tang, C. J. Van Westen, H. Tanyas, V. G. Jetten, Analysing post-earthquake landslide activity using multi-temporal landslide inventories near the epicentral area of the 2008 Wenchuan earthquake. Nat. Hazards Earth Syst. Sci. 16, 2641–2655 (2016).

[40] H. Tanyaş, D. Kirschbaum, T. Görüm, C. J. van Westen, C. Tang, L. Lombardo, A closer look at factors governing landslide recovery time in post-seismic periods. Geomorphology 391, 107912 (2021).

[41] Culmann, C., Graphische Statik (Meyer and Zeller, Zurich, Switzerland, 1875).

[42] N. Lu, J. W. Godt, *Hillslope Hydrology and Stability* (Cambridge Univ. Press, Cambridge, 2013).

[43] J. W. Godt, R. L. Baum, N. Lu, Landsliding in partially saturated materials. Geophys. Res. Lett. 36, 10.1029/2008GL035996 (2009).

[44] K. M. Schmidt, D. R. Montgomery, Limits to relief. Science 270, 617–620 (1995).

[45] G. M. Belair, J. M. Jones, S. N. Martinez, B. B. Mirus, N. J. Wood, “Slope-relief threshold landslide susceptibility models for the United States and Puerto Rico, U.S. Geological Survey” (2024); 10.5066/P13KAGU3.

[46] G. L. Bennett, S. R. Miller, J. J. Roering, D. A. Schmidt, Landslides, threshold slopes, and the survival of relict terrain in the wake of the Mendocino Triple Junction. Geology 44, 363–366 (2016).

[47] B. Campforts, C. M. Shobe, I. Overeem, G. E. Tucker, The art of landslides: How stochastic mass wasting shapes topography and influences landscape dynamics. J. Geophys. Res. Earth 127, e2022JF006745 (2022).

[48] A. L. Densmore, M. A. Ellis, R. S. Anderson, Landsliding and the evolution of normal-fault-bounded mountains. J. Geophys. Res. Solid Earth 103, 15203–15219 (1998).

[49] R. A. DiBiase, M. W. Rossi, A. B. Neely, Fracture density and grain size controls on the relief structure of bedrock landscapes. Geology 46, 399–402 (2018).

[50] L. Jeandet, P. Steer, D. Lague, P. Davy, Coulomb mechanics and relief constraints explain landslide size distribution. Geophys. Res. Lett. 46, 4258–4266 (2019).

[51] D. R. Montgomery, M. T. Brandon, Topographic controls on erosion rates in tectonically active mountain ranges. Earth Planet. Sci. Lett. 201, 481–489 (2002).

[52] K. F. Townsend, S. F. Gallen, M. K. Clark, Quantifying near-surface rock strength on a regional scale from hillslope stability models. J. Geophys. Res. Earth 125, e2020JF005665 (2020).

[53] B. B. Mirus, G. M. Belair, N. J. Wood, J. Jones, S. N. Martinez, Parsimonious high-resolution landslide susceptibility modeling at continental scales. AGU Adv. 5, e2024AV001214 (2024).

[54] U.S. Geological Survey, “Shuttle Radar Topography Mission 1 Arc-Second Global” (2018); 10.5066/F7PR7TFT.

[55] M. E. A. Budimir, P. M. Atkinson, H. G. Lewis, A systematic review of landslide probability mapping using logistic regression. Landslides 12, 419–436 (2015).

[56] M. Alvioli, M. Loche, L. Jacobs, C. H. Grohmann, M. T. Abraham, K. Gupta, N. Satyam, G. Scaringi, T. Bornaetxea, M. Rossi, I. Marchesini, L. Lombardo, M. Moreno, S. Steger, C. A. S. Camera, G. Bajni, G. Samodra, E. E. Wahyudi, N. Susyanto, M. Sinčić, S. B. Gazibara, F. Sirbu, J. Torizin, N. Schüßler, B. Mirus, J. Woodard, H. Aguilera, J. S. Rivera-Rivera, A benchmark dataset and workflow for landslide susceptibility zonation. Earth Sci. Rev. 258, 104927 (2024).

[57] E. R. C. Baynes, D. Lague, M. Attal, A. Gangloff, L. A. Kirstein, A. J. Dugmore, River self-organisation inhibits discharge control on waterfall migration. Sci. Rep. 8, 2444 (2018). 29402911 10.1038/s41598-018-20767-6 PMC5799191

[58] A. Bigi, L. E. Hasbargen, A. Montanari, C. Paola, Knickpoints and hillslope failures: Interactions in a steady-state experimental landscape. Spec. Pap.Geol. Soc. Am. 398, 295 (2006).

[59] K. Koshimizu, S. Ishimaru, F. Imaizumi, G. Kawakami, Morphological characteristics and conditions of drainage basins contributing to the formation of debris flow fans: Examination of regions with different rock strength using decision tree analysis. Nat. Hazards Earth Syst. Sci. Discuss. 24, 1287–1301 (2024).

[60] R. Santos, R. M. Duarte, Topographic signature of debris flow dominated channels: Implications for hazard assessment. WIT Trans. Ecol. Environ. 90, 301–310 (2006).

[61] L. A. Richards, Capillary conduction of liquids through porous mediums. Phys. Ther. 1, 318–333 (1931).

[62] A. Golly, J. M. Turowski, A. Badoux, N. Hovius, Controls and feedbacks in the coupling of mountain channels and hillslopes. Geology 45, 307–310 (2017).

[63] R. Emberson, D. Kirschbaum, P. Amatya, H. Tanyas, O. Marc, Insights from the topographic characteristics of a large global catalog of rainfall-induced landslide event inventories. Nat. Hazards Earth Syst. Sci. Discuss. 22, 1129–1149 (2021).

[64] O. Marc, M. Gosset, H. Saito, T. Uchida, J.-P. Malet, Spatial patterns of storm-induced landslides and their relation to rainfall anomaly maps. Geophys. Res. Lett. 46, 11167–11177 (2019).

[65] S. Peruccacci, M. T. Brunetti, S. L. Gariano, M. Melillo, M. Rossi, F. Guzzetti, Rainfall thresholds for possible landslide occurrence in Italy. Geomorphology 290, 39–57 (2017).

[66] R. C. Wilson, A. S. Jayko, “Preliminary maps showing rainfall thresholds for debris-flow activity, San Francisco Bay region, California” (Open File Report 97-745-F, U.S. Geological Survey, 1997); 10.3133/ofr97745F.

[67] E. E. Brabb, J. P. Colgan, T. C. Best, “Map showing inventory and regional susceptibility for Holocene debris flows, and related fast-moving landslides in the conterminous United States: U.S. Geological Survey miscellaneous field studies map 2329 (1999); https://pubs.usgs.gov/mf/1999/2329/.

[68] N. K. Meyer, W. Schwanghart, O. Korup, B. Romstad, B. Etzelmüller, Estimating the topographic predictability of debris flows. Geomorphology 207, 114–125 (2014).

[69] D. G. Milledge, A. L. Densmore, D. Bellugi, N. J. Rosser, J. Watt, G. Li, K. J. Oven, Simple rules to minimise exposure to coseismic landslide hazard. Nat. Hazards Earth Syst. Sci. 19, 837–856 (2019).

[70] J. D. Stock, W. E. Dietrich, Valley incision by debris flows: Evidence of a topographic signature. Water Resour. Res. 39, 10.1029/2001WR001057 (2003).

[71] R. Strauch, E. Istanbulluoglu, S. S. Nudurupati, C. Bandaragoda, N. M. Gasparini, G. E. Tucker, A hydroclimatological approach to predicting regional landslide probability using Landlab. Earth Surf. Dyn. 6, 49–75 (2018).

[72] D. L. Turcotte, *Fractals and Chaos in Geology and Geophysics* (Cambridge Univ. Press, New York, 2012).

[73] W. R. Tobler, A computer movie simulating urban growth in the Detroit region. Econ. Geogr. 46, 234–240 (1970).

[74] E. E. Brabb, Innovative approaches to landslide hazard and risk mapping, in *4th International Symposium on Landslides* (Toronto, 1984) vol. 1, pp. 307–324.

[75] U.S. Geological Survey, 3D Elevation Program 1/3 arcsecond (2019); https://apps.nationalmap.gov/downloader/.

[76] Oregon Department of Geology and Mineral Industries [DOGAMI], Statewide Landslide Information Database for Oregon [SLIDO], version 4.5 (2024); https://www.oregon.gov/dogami/slido/Pages/data.aspx.

[77] G. King, L. Zeng, Explaining rare events in international relations. Int. Organ. 55, 693–715 (2001).

[78] T. Oommen, L. G. Baise, R. M. Vogel, Sampling bias and class imbalance in maximum-likelihood logistic regression. Math. Geosci. 43, 99–120 (2011).

[79] Y. Rubner, C. Tomasi, L. J. Guibas, A metric for distributions with applications to image databases, in *Sixth International Conference on Computer Vision* (*IEEE Cat. No.98CH36271*, 1998), pp. 59–66.

[80] G. Jia, M. Alvioli, S. L. Gariano, I. Marchesini, F. Guzzetti, Q. Tang, A global landslide non-susceptibility map. Geomorphology 389, 107804 (2021).

[81] J. W. Godt, J. A. Coe, R. L. Baum, L. M. Highland, J. R. Keaton, R. J. R. Jr., Prototype landslide hazard map of the conterminous United States (2012), pp. 245–250.

[82] I. Marchesini, F. Ardizzone, M. Alvioli, M. Rossi, F. Guzzetti, Non-susceptible landslide areas in Italy and in the Mediterranean region. Nat. Hazards Earth Syst. Sci. 14, 2215–2231 (2014).

[83] G. M. Belair, E. S. Jones, S. L. Slaughter, B. B. Mirus, “Landslide inventories across the United States version 2” (2022); 10.5066/P9FZUX6N.

[84] H. Tanyas, M. Rossi, M. Alvioli, C. J. van Westen, I. Marchesini, A global slope unit-based method for the near real-time prediction of earthquake-induced landslides. Geomorphology 327, 126–146 (2019).

[85] A. R. R. Grant, W. T. Struble, S. R. LaHusen, Limits to coseismic landslides triggered by Cascadia Subduction Zone earthquakes. Geomorphology 418, 108477 (2022).

[86] A. Merghadi, A. P. Yunus, J. Dou, J. Whiteley, B. ThaiPham, D. T. Bui, R. Avtar, B. Abderrahmane, Machine learning methods for landslide susceptibility studies: A comparative overview of algorithm performance. Earth Sci. Rev. 207, 103225 (2020).

[87] J. J. Roering, B. H. Mackey, A. L. Handwerger, A. M. Booth, D. A. Schmidt, G. L. Bennett, C. Cerovski-darriau, Beyond the angle of repose: A review and synthesis of landslide processes in response to rapid uplift, Eel River, North California. Geomorphology 236, 109–131 (2015).

[88] Biden, Joseph R., “Executive Order On Advancing Racial Equity and Support for Underserved Communities Through the Federal Government” (2021); https://www.whitehouse.gov/briefing-room/presidential-actions/2021/01/20/executive-order-advancing-racial-equity-and-support-for-underserved-communities-through-the-federal-government/; https://www.federalregister.gov/documents/2021/01/25/2021-01753/advancing-racial-equity-and-support-for-underserved-communities-through-the-federal-government).

[89] U.S. Geological Survey, “USGS National Hydrography Dataset Plus High Resolution” (2019); https://www.sciencebase.gov/catalog/item/57645ff2e4b07657d19ba8e8.

[90] J. Stoker, B. Miller, The accuracy and consistency of 3D elevation program data: A systematic analysis. Remote Sens 14, 940 (2022).

[91] R. B. Moore, L. D. McKay, A. H. Rea, T. R. Bondelid, C. V. Price, T. G. Dewald, C. M. Johnston, “User’s guide for the national hydrography dataset plus (NHDPlus) high resolution” (Open File Report 2019-1096, U.S. Geological Survey, 2019); 10.3133/ofr20191096.

[92] GRASS Development Team, “Geographic Resources Analysis Support System (GRASS) Software, version 7.8, Open Source Geospatial Foundation” (2020); https://grass.osgeo.org.

[93] R Core Team, R: A language and environment for statistical computing (2022).

[94] J. T. Falgout, J. Gordon, L. Lee, B. Williams, “USGS Advanced Research Computing, USGS Hovenweep Supercomputer, U.S. Geological Survey” (2023); 10.5066/P927BI7R.

[95] S. Slaughter, W. Burns, K. Mickelson, K. Jacobacci, A. Biel, T. Contreras, Protocol for landslide inventory mapping from lidar data in Washington State. Wash. Geol. Surv. Bull. 82, 3 (2017).

[96] Washington Geological Survey, Washington State Landslide Inventory Database-GIS data, version 1.2 (2023); https://fortress.wa.gov/dnr/geologydata/publications/data_download/ger_portal_landslide_database.zip.

[97] W. J. Burns, I. P. Madin, “Protocol for inventory mapping of landslide deposits from light detection and ranging (LiDAR) imagery” (Oregon Department of Geology and Mineral Industries, 2009); https://pubs.oregon.gov/dogami/dds/slido/sp-42_onscreen.pdf.

[98] W. J. Burns, N. C. Calhoun, J. J. Franczyk, “Protocol for channelized debris flow susceptibility mapping” (Oregon Department of Geology and Mineral Industries, 2022); https://pubs.oregon.gov/dogami/sp/SP-53/SP-53_report.pdf.

[99] R. J. Hofmeister, “Slope failures in Oregon GIS inventory for three 1996/97 storm events” (Oregon Department of Geology and Mineral Industries, 2000); https://pubs.oregon.gov/dogami/sp/p-SP-34.htm.

[100] M. Loche, M. Alvioli, I. Marchesini, H. Bakka, L. Lombardo, Landslide susceptibility maps of Italy: Lesson learnt from dealing with multiple landslide types and the uneven spatial distribution of the national inventory. Earth Sci. Rev. 232, 104125 (2022).

[101] L. Jacobs, M. Kervyn, P. Reichenbach, M. Rossi, I. Marchesini, M. Alvioli, O. Dewitte, Regional susceptibility assessments with heterogeneous landslide information: Slope unit- vs. pixel-based approach. Geomorphology 356, 107084 (2020).

[102] B. McCune, D. Keon, Equations for potential annual direct incident radiation and heat load. J. Veg. Sci. 13, 603–606 (2002).

[103] S. Lloyd, Least squares quantization in PCM. IEEE Trans. Inf. Theory 28, 129–137 (1982).

[104] A. Brenning, “Spatial cross-validation and bootstrap for the assessment of prediction rules in remote sensing: The R package sperrorest,” in *2012 IEEE International Geoscience and Remote Sensing Symposium* (IEEE, 2012), pp. 5372–5375.

[105] ESRI, World Hillshade (2023); https://services.arcgisonline.com/arcgis/rest/services/Elevation/World_Hillshade/MapServer.

[106] ESRI, World Oceans Basemap, (2024); https://services.arcgisonline.com/ArcGIS/rest/services/Ocean/World_Ocean_Base/MapServer.

[107] J. B. Woodard, B. B. Mirus, “Morphometric landslide susceptibility results of the Northwestern United States and Southwestern Canada derived from elevation data” (2024); 10.5066/P13AXWAA.

